# High-Dose Neural Stem/Progenitor Cell Transplantation Increases Engraftment and Neuronal Distribution and Promotes Functional Recovery in Rats after Acutely Severe Spinal Cord Injury

**DOI:** 10.1155/2019/9807978

**Published:** 2019-09-02

**Authors:** Taoyang Yuan, Qian Liu, Jie Kang, Hua Gao, Songbai Gui

**Affiliations:** ^1^Department of Neurosurgery, Beijing Tiantan Hospital, Capital Medical University, Beijing, China; ^2^Beijing Neurosurgical Institute, Capital Medical University, Beijing, China

## Abstract

Severe spinal cord injury (SCI) leads to permanent, complete paraplegia and places considerable mental and economic burdens on patients, compared with mild to moderate SCI. However, the dose-related effects of the neural stem/precursor cell (NSPC) transplantation on the injury microenvironment, NSPC survival, axonal growth, neuronal distribution, the composition of neurons, oligodendrocytes, and astrocytes in the lesion area and functional recovery have not yet been quantitatively evaluated in the context of severe SCI. In our study, we acutely transplanted 2.5 × 10^4^ or 1.5 × 10^5^ NSPCs/*μ*l into the site of transection SCI. We found that high-dose NSPC transplantation exerted immunomodulatory and neuroprotective effects in the acute phase of severe SCI. In addition, one week later, a remarkable positive relationship was observed between the transplantation dose and the number of surviving NSPCs in severe SCI. At 8 weeks postgrafting, subjects that received the higher cell dose exhibited abundant nerve regeneration, extensive neuronal distribution, increased proportions of neurons and oligodendrocytes, and nascent functional neural network formation in the lesion area. Notably, a significant functional recovery was also observed. Our data suggest that it is important to consider potential dose-related effects on donor cell survival, neuronal distribution, and locomotor recovery in the development of preclinical NSPC transplantation therapy for severe SCI.

## 1. Introduction

Spinal cord injury (SCI) leads to permanent, complete paraplegia and places considerable mental and economic burdens on patients, especially severe SCI, which causes extensive neuronal loss, acute axonal damage, demyelination, and scar formation [1, 2]. Currently, there is no effective treatment for the injured spinal cord in the clinic [3, 4]. The transplantation of Schwann cells, neural stem /progenitor cells (NSPCs), embryonic stem cells, Induced Pluripotent Stem Cells (IPSCs), and Mesenchymal Stem Cells (MSCs) has been investigated in an animal model as potential therapies for spinal cord injury [5]. In these cells, neural stem/progenitor cells (NSPCs) are considered a promising therapeutic strategy for spinal cord injury (SCI) because of NSPCs isolated from the central nervous system and often grown as neurospheres with the capacity to differentiate into neurons and glia, thus, have the potential to not only replace lost neurons and glia but also restore disrupted connectivity at the lesion site [5–9]. In recent years, many studies have reported that the transplantation of NSPCs from different sources promotes functional recovery after mild and moderate SCI in rodents. Potential mechanisms include the following: (i) NSPCs differentiate into neurons to replace lost neural tissue at the injury site [10–13]; (ii) NSPCs secrete neurotrophic factors to improve the microenvironment of the injury site [13–15]; (iii) NSPCs promote axonal regeneration and remyelination [16–18]; and (iv) notably, NSPCs form a nascent functional neural network to deliver signals [12, 19, 20]. But only a few studies have investigated the effects of NSPCs in severe SCI, and their results have suggested that NSPC transplantation have limited ability to promote functional recovery in severe SCI [19, 21, 22]. The factors of this finding include the poor survival of grafted NSPCs which was caused by the absence of an extracellular matrix to support grafted NSPCs as well as a harmful environment formed at the injury site [21, 23]. Furthermore, most of the surviving grafted NSPCs differentiated into astrocytes and oligodendrocytes, with the development of only a few neurons which play an important role in motor functional recovery following severe SCI [24–26]. Efforts have been made to overcome this limitation by altering the time and site of NSPC transplantation. Li et al. investigated NSC transplantation in the spinal cord rostral/caudal to the transection site at the subacute stage, compared with those rostral/caudal to the transection site at the acute stage [27]. Recently, Piltti et al. reported that the transplantation dose alters the dynamics of human NSPC engraftment, proliferation, and migration, as well as oligodendroglial and neuronal differentiation, in the early chronic stage 30 days after moderate thoracic SCI [1, 28]. However, no studies have yet quantitatively investigated the effects of different transplantation doses on the NSPC survival, the injury microenvironment, the neuronal distribution, or the functional recovery in the acute phase of severe SCI in rats.

In our study, we investigated whether the dose of transplanted NSPCs affects the early survival of grafted NSPCs at 1 week postsurgery in rats and whether the dose of the transplanted NSPCs can affect the harmful microenvironment following acute transplantation in severe SCI. Then, we also observed the long-term effects of different transplantation dose on the neuronal distribution, the axonal regeneration, the repair of tissue structure in the lesion area, and the functional recovery after severe SCI at 8 weeks postsurgery.

## 2. Materials and Methods

### 2.1. The Isolation and Culture of Rat NSPCs

The methods for the culture and expansion of NSPCs have been previously described [29]. In brief, E18 cerebral anlage tissue from transgenic GFP-expressing Sprague-Dawley (SD) rats was mechanically dissociated in basal medium (DMEM/F12). Then, the dissociated cells were collected by centrifugation, and the cell suspension was cultured in DMEM containing 1% B27 (Invitrogen), basic fibroblast growth factor (bFGF, 20 ng/ml; Invitrogen), epidermal growth factor (EGF, 20 ng/ml; Invitrogen), and N2 (10 *μ*l/ml; Invitrogen) ([Supplementary-material supplementary-material-1]). On the day of transplantation, to identify NSPCs in vitro, the cells from passage 3 ([Supplementary-material supplementary-material-1]) were immunostained with Nestin, the result showed strong and stable emission of the green fluorescent signal and formed neurospheres ([Supplementary-material supplementary-material-1]), and the neurospheres expressed Nestin ([Supplementary-material supplementary-material-1]). To observe the differentiation potential of the NSPCs, the cells at passage 3 were cultured in a medium containing 1% fetal bovine serum without EGF or bFGF. We detected the cells expressing the neuronal marker MAP-2 ([Supplementary-material supplementary-material-1]), the astrocytic marker glial fibrillary acidic protein (GFAP) ([Supplementary-material supplementary-material-1]), and the oligodendrocyte marker O1 ([Supplementary-material supplementary-material-1]) seven days later. This result indicated that the transplanted NSPCs have the capacity to differentiate to neurons, astrocytes, and oligodendrocytes. Before transplantation, the NSPCs were prepared as 1.5 × 10^5^ viable cells per microliter and 2.5 × 10^4^ viable cells per microliter in 0.1 M PBS after digestion with 0.05% trypsin/DMEM and washing with basal medium (DMEM/F12).

### 2.2. Spinal Cord Injury and Transplantation

Healthy adult female SD rats (220-250 g, *n* = 85) purchased from Beijing Vital River Laboratory Animal Technology Corporation were used in this study. All experimental protocols and animal handling procedures were approved by the Beijing Neurosurgical Institute Laboratory Animal Ethics Committee in China. All surgeries were performed under anesthesia with intraperitoneal (i.p.) injections of 10% chloral hydrate (0.3 ml/100 g of body weight). After inducing deep anesthesia and incising the skin, subcutaneous tissue, and muscle, a laminectomy was performed to expose the T10 spinal cord region under an operating microscope. The dura was cut with an 11-blade scalpel, and then, a 2 mm long section of the spinal cord was completely removed at the T10 level using iridectomy scissors and microscope forceps under the dura. The operating board was spun, and the operating microscope was adjusted to clear residual fibers. After complete SCI, 10 *μ*l of cell suspension (1.5 × 10^5^ viable cells per microliter in the high-dose group, *n* = 30; 2.5 × 10^4^ viable cells per microliter in the low-dose group, *n* = 30) was immediately microinjected into the lesion cavity using a Hamilton syringe. Subjects injected with 10 *μ*l of PBS were used as the operation control (SCI group, *n* = 25). After the surgical incisions were sutured, the subjects were placed on a heating pad until fully awake and then received 0.9% saline i.p. (3 ml per subject). Postoperative care included the administration of penicillin once a day for three days to prevent infection and manual pressing of the bladder twice a day until recovery of automatic micturition. The subjects that underwent NSPC transplantation were not immunosuppressed due to the homogeneity with the NSPCs, and the NSPC-transplanted rats did not incur notable immune responses compared to those in the control group throughout the entire experiment.

### 2.3. Behavioral Analysis

Locomotor function was evaluated using the Basso-Beattie-Bresnahan (BBB) locomotor rating scale [30]. The open-field environment was a molded plastic wading pool with a nonslippery surface (100 cm in diameter and 21 cm in height) [30]. The BBB scores were assessed weekly after the surgery. Seven subjects were randomly selected for testing, which lasted for approximately 4 min and was recorded using a digital video camera (Sony). The hindlimb movement scores were assessed by two independent observers blinded to the group identities.

### 2.4. Electrophysiology

Motor potentials were evoked and recorded according to a previously described protocol [31, 32]. The main difference in our procedure was the i.p. injection of 10% chloral hydrate (0.3 ml/100 g of body weight) [33]. To record the motor-evoked potentials (MEPs), one needle electrode was placed in the tibialis anterior muscle (the cathode) and another was placed subcutaneously at the level of the foot pad (the anode). To induce the MEPs (after central stimulation), one needle electrode was placed subcutaneously at the level of the lower jaw (the anode) and another was placed at the cranial level (the cathode). For the ground, an electrode was placed subcutaneously in the lumbar region. The electrophysiological signals were recorded by an electromyography system (Neuro-MEP-Micro, Russia) with bandpass filtering from 2 Hz to 10 kHz. The pulse duration used throughout the experiments was 0.1 ms. To induce the MEPs, stimulation with an intensity of 25 mA was applied to the needle electrode at the cranial level. MEP measurements were performed in the three groups at 8 weeks after the operation (*n* = 7 animals per group).

### 2.5. Neuronal Tracing

To label the corticospinal tract (CST) fibers, 4 *μ*l of biotinylated dextran amine (BDA, 10%, 10,000 MW; Invitrogen) was bilaterally injected into the motor cortices at eight sites (0.5 *μ*l per site) at 8 weeks postsurgery (*n* = 3 per group) [34]. Two weeks later, the subjects were perfused with 4% paraformaldehyde (PFA). To trace the functional transsynaptic neural pathways by labeling the descending CST fibers, 1% biotinylated cholera toxin B subunit (CTB, Invitrogen, C-34779, Molecular Probes), which can transfer across synapses to second- and third-order neurons [35, 36], was injected into bilateral L2/L3 spinal white matter using a Hamilton syringe (1 *μ*l per site) in three subjects from each group. Seven days postinjection, the subjects were perfused with 4% PFA, and the spinal cord was subjected to CTB staining.

### 2.6. Histology and Immunohistochemistry

After being deeply anesthetized, all animals were perfused with 4% PFA, and spinal cord segments containing a lesion or graft site were obtained. The samples were placed in 4% PFA overnight at 4°C and then transferred to 30% sucrose solution (with 0.1 M PBS) and incubated overnight. The spinal cord samples were embedded in OCT compound and frozen in n-hexane (-80°C) after being processed. Horizontal cryosections, 10 *μ*m thick, were cut using a cryostat microtome (Leica). The sections were washed three times with 0.01 M PBS and then incubated with the primary antibody chicken anti-GFP (1 : 200, Thermo Fisher Scientific, to label GFP-expressing NSPCs), rabbit anti-MAP-2 (1 : 50, CST, to label mature neurons), rabbit anti-NeuN (1 : 200, Abcam, to label mature neurons), mouse anti-*β*III tubulin (Tuj1, 1 : 200, CST, to label immature and mature neurons), mouse anti-O1 (1 : 100, Millipore, to label oligodendrocytes), rabbit antisynaptophysin (SYN, 1 : 200, CST, to label presynaptic terminals), rabbit antineurofilament (NF, 1 : 1000, Abcam, to label axons), mouse anti-GFAP (1 : 300, CST, to label astrocytes), mouse anti-MBP (1 : 200, Abcam, to label oligodendrocytes and myelin), and mouse anticholine acetyltransferase (CHAT, 1 : 200, Abcam, to label spinal cord motor neurons and motor axons) overnight at 4°C. Then, after being washed three times with 0.01 M PBS, the samples were incubated for 2 hours at 4°C with Alexa Fluor 488-, 594-, and 647-conjugated goat antibodies (1 : 250, Thermo Fisher Scientific) or Alexa Fluor 594-conjugated streptavidin (1 : 250, Invitrogen) to label BDA-traced CST axons or CTB-traced neurons. After the sections were washed again with 0.01 M PBS, mounting medium with DAPI (nuclear staining; ZSGB-BIO, China) was added to the sections, which were then cover slipped. All samples were permeabilized with 0.03% Triton X-100 and blocked for 1 hour at room temperature with 10% normal horse serum. Finally, sections were examined and imaged by confocal laser scanning microscopy (Zeiss, Airyscan LSM880, Germany).

### 2.7. Quantitative Immunohistochemical Analyses

To quantitatively analyze the survival of GFP-positive grafted cells in the lesion area, three subjects each were sacrificed in the high- and low-dose groups at 1 week postgrafting. Then, one for every ten in the whole series of sections from each rat was selected for immunostaining with GFP and DAPI. One random image within the rostral, central, and caudal areas of the SCI site was, respectively, imaged in each section at 400x magnification using an LSM880 system. Finally, the numbers of GFP-positive cells in each experiment rat were quantified using the Image-Pro Plus software (Media Cybernetics, Silver Spring, MD).

To quantify axonal regeneration in vivo, four rats were sacrificed in every group, and one for every ten in the whole series of sections from each rat was selected. After all the selected sections were immunolabeled for NFs, one random image within the rostral, central, and caudal areas of the SCI graft site was, respectively, imaged in each section at 200x magnification using an LSM880 system. Then, the number of NF-positive axons in every image was quantified using the Image-Pro Plus software (Media Cybernetics, Silver Spring, MD). In brief, an image was chosen, and NF-positive nerve fibers were converted into an area of interest (AOI); then, the pixel area of the AOI was automatically calculated by the software [37, 38]. Finally, we statistically analyzed the area of NF-positive fibers in the rostral, central, and caudal regions of the lesion site in each rat from the three groups (*n* = 4 per group).

To quantitatively analyze the area of MAP-2-positive neurons in the lesion core, one random area in the central region of the SCI graft site was, respectively, imaged in each section at 200x magnification using an LSM880 system (ten sections per rat, *n* = 4 per group). The Image-Pro Plus software was used as described above to evaluate the proportion of MAP-2-positive neurons in the lesion core.

To quantify the percentage of cells that were positive for GFAP, MAP-2, and MBP in the regenerated tissue at the SCI (2 mm) site at 8 weeks postsurgery, three sequential sections for every ten in the whole series of sections from each animal were selected for GFAP, MAP-2, and MBP immunostaining (*n* = 4 rats per group). One random area within the rostral, central, and caudal regions of the SCI graft site was, respectively, imaged in each section at 200x magnification using an LSM880 system. We assessed the mean area of cells positive for GFAP, MAP-2, and MBP in each section using the Image-Pro Plus software, as described above. Then, we quantitatively analyzed the area of cells positive for each marker in the total area of regenerated tissue, i.e., the sum of the areas stained positive for GFAP, MAP-2, and MBP, in one rat.

### 2.8. RNA Isolation and RT-PCR

To compare the mRNA expression levels of the three groups at 1, 3, and 7 days postsurgery (*n* = 3 per group), total RNA was extracted from 3 mm long spinal cord samples, which included the lesion area, using the TRIzol Reagent (Life Technologies). To synthesize complementary DNA (cDNA), reverse transcription was performed using a High-Capacity cDNA Reverse Transcription kit (Life Technologies) according to the manufacturer's instructions. The gene expression levels are listed in supplemental Table 1. Gene expression was quantified using an Applied Biosystems 7500 Fast System (Life Technologies). The data are presented as fold-change values.

### 2.9. Transmission Electron Microscopy (TEM)

To detect remyelination and axonal regeneration, subjects (*n* = 3 per group) randomly selected from each experiment group were perfused with 4% PFA at 8 weeks postsurgery. Then, the samples containing a lesion or graft site were obtained and postfixed with 1% osmium tetroxide, dehydrated, and embedded in Durcupan resin. To obtain axial ultrathin sections in the lesion area, the samples were cut from the lesion core using an ultramicrotome (Leica EM UC7). Then, axial ultrathin sections were examined under a transmission electron microscope (HITACHI, H-7650, Japan).

### 2.10. Statistical Analysis

All statistical analyses were performed using the GraphPad Prism 6.0. All data are presented as the mean ± SEM. Correlations between the transplantation dose and the number of surviving NSPCs were assessed using the Pearson correlation coefficients and linear regression analyses. One-way ANOVA with Fisher's least significant difference (LSD) test was used to assess the comparisons among the three groups. If equal variances were found, the LSD test was applied; otherwise, the Kruskal-Wallis test and Dunnett's T3 test were used. Two-group comparisons were made by using Student's *t*-tests. The significance level was set at *p* < 0.05.

## 3. Results

### 3.1. High-Dose NSPC Transplantation Improved the Injury Microenvironment and Significantly Increased NSPC Survival and Integration

**Figure 1:**
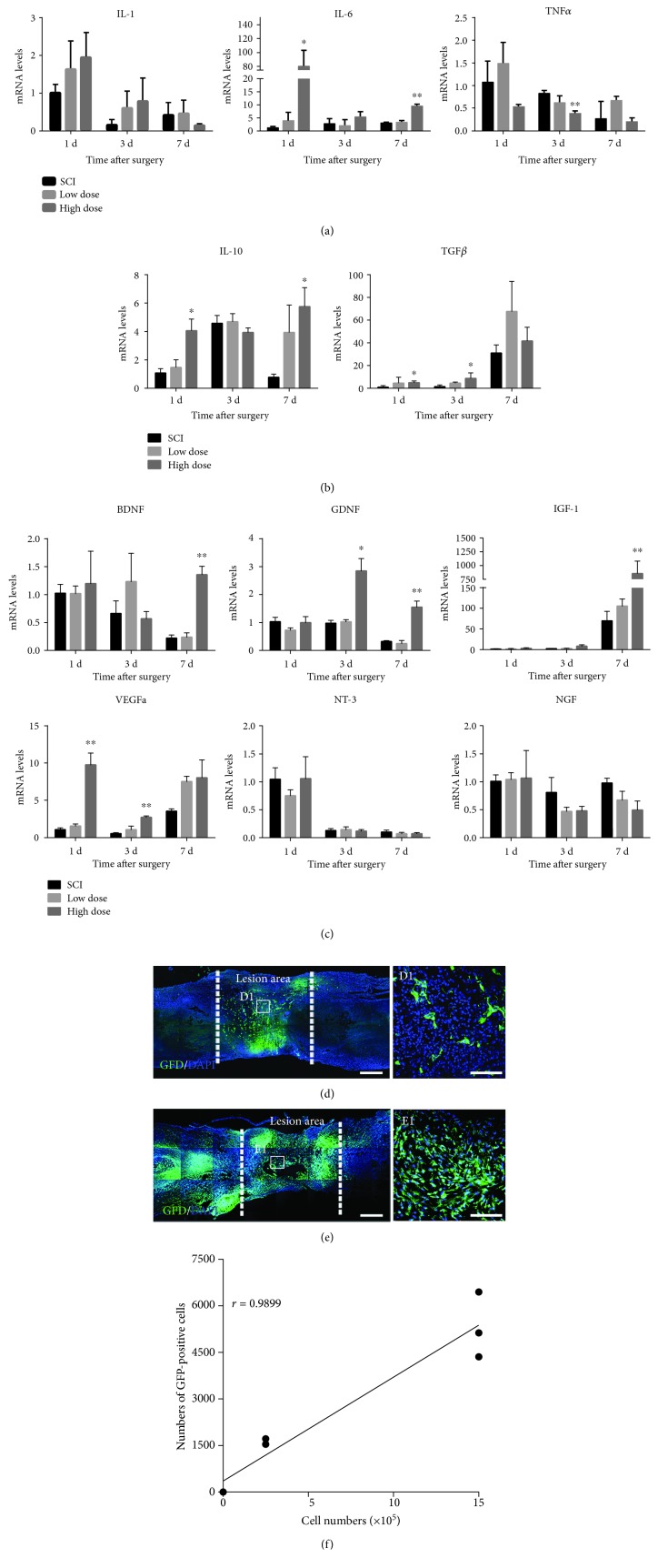
High-dose transplantation improved injury microenvironment and significantly increased survival of NSPCs. (a) The mRNA levels of proinflammatory cytokines between the SCI, low-dose, and high-dose group (*n* = 3 animals per group, ^∗^*p* < 0.05 versus the SCI group; data are represented as mean ± SEM). (b) The mRNA expression of anti-inflammatory cytokines was measured by quantitative RT-PCR at three groups (*n* = 3 animals per group). There was a significant increase of anti-inflammatory cytokines in the high-dose group compared with the SCI group, not the low-dose group (^∗^*p* < 0.05 versus the SCI group; data are represented as mean ± SEM). (c) Time course of the gene expressions of neurohumoral factors in three groups (*n* = 3 animals per group). The mRNA level of BDNF, GDNF, IGF-1, and VEGF-A significantly increase at different time post high-dose transplantation compared to the SCI group (^∗^*p* < 0.05, ^∗∗^p < 0.01 versus the SCI group; data are represented as mean ± SEM). (d, e) Immunohistochemical analysis of engrafted NSPCs at 7 days postgrafting in the high-dose and low-dose group (the white dotted lines indicated the boundary between the host spinal cord and the lesion area). Scale bars: 500 *μ*m. (D1, E1) High magnifications from (d, e) show GFP-positive cells survive in the lesion area. Scale bars: 100 *μ*m. (f) Correlation between the numbers of surviving GFP-positive cells and the transplanted dose at 7 days postgrafting (Pearson *r* = 0.9899, *p* < 0.05, *n* = 3 animals per group).

In this study, we used qPCR to examine whether the number of transplanted cells affects the harmful microenvironment; different numbers of NSPCs were transplanted into the SCI site immediately after complete injury at the T10 level. High-dose NSPC transplantation notably increased the expression of anti-inflammatory cytokines, such as interleukin- (IL-) 10 and transforming growth factor- (TGF-) *β*, in addition to attenuating the expression level of tumor necrosis factor- (TNF-) *α*, at 3 days postsurgery (Figures 1(a) and 1(b)). Furthermore, in the high-dose group, we observed significant increases in the expression levels of several neurotrophic factors, including glial cell-derived neurotrophic factor (GDNF), brain-derived neurotrophic factor (BDNF), insulin-like growth factor- (IGF-) 1, and vascular endothelial growth factor- (VEGF-) A but not neurotrophic factor- (NT-) 3 or nerve growth factor (NGF) (Figure 1(c)). However, there were no significant differences in the cytokine mRNA expression levels between the low-dose and SCI groups.

Next, we conducted immunofluorescence analysis to examine whether high-dose NSPC transplantation enhanced the engraftment and survival of NSPCs in severe SCI to complement the apoptosis and loss of grafted cells. Immunostaining showed greater GFP-positive cell survival and integration in the lesion area in the high-dose group than in the low-dose group and more grafted cells distributed in the rostral and caudal areas of the lesion and migrated into the host spinal cord in the high-dose group (Figures 1(d) and 1(e)). The Pearson correlation analysis revealed a remarkable positive relationship between the transplantation dose and the number of surviving NSPCs after severe SCI at 7 days postgrafting (Figure 1(f), *r* = 0.9899).

### 3.2. High-Dose NSPC Transplantation Significantly Promoted Motor Functional Recovery and Electrophysiological Recovery in Rats with Complete SCI

**Figure 2:**
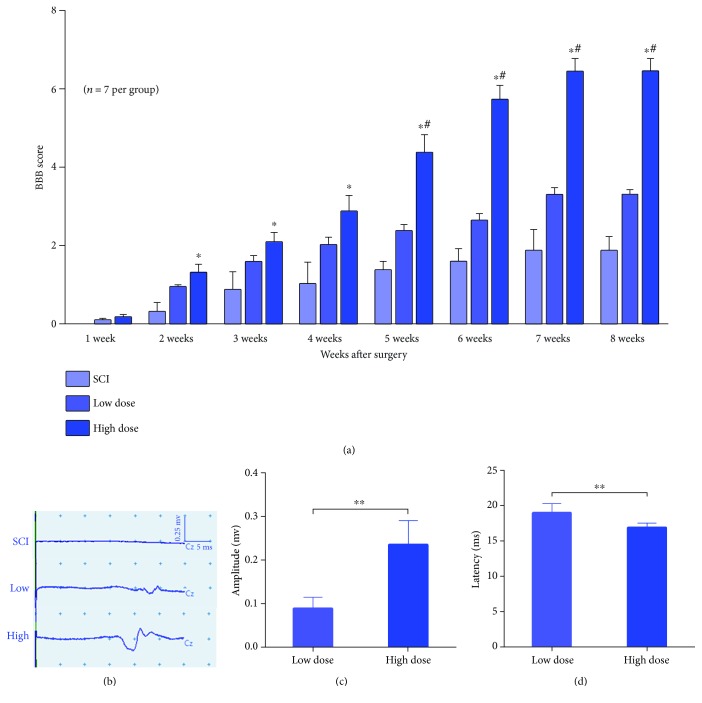
High-dose NSPC transplantation significantly promoted motor functional and electrophysiological recovery in rats with complete SCI. (a) The locomotor function of the hindlimbs was assessed by the Basso, Beattie, Bresnahan (BBB); the high-dose group exhibited significantly greater motor functional recovery compared to the SCI groups at 2 weeks postgrafting. Besides, the high-dose group exhibited significantly greater motor functional recovery compared to the low-dose group after 5 weeks postgrafting (mean ± SEM, *n* = 7 per group, ^∗^*p* < 0.05, high-dose group versus SCI group, ^#^*p* < 0.05, high-dose group versus low-dose group). (b) 8 weeks postgrafting, the electrophysiological signal appeared in the high- and low-dose group but not in the control animals. (c, d) Amplitude and latency of MEP response in the high- and low-dose groups. The high-dose group showed a significantly higher MEP amplitude than the low-dose group, and the MEP latency was significantly longer in the low-dose group than in the high-dose group (mean ± SEM, *n* = 7 animals per group; ^∗∗^*p* < 0.01).

To examine the influence of the NSPC transplantation dose on functional recovery, we quantitatively evaluated functional improvements every week after surgery using the BBB open-field locomotor scale in subjects with complete SCI at the T10 level treated with either low- or high-dose NSPC transplantation. Two weeks postsurgery, we found greater hindlimb locomotion recovery in the high-dose groups than in the SCI group; there were no differences between the high- and low-dose groups (Figure 2(a)). At five weeks postsurgery, a significant improvement in the BBB scores was observed in the high-dose group compared with the low-dose group (Figure 2(a)), and a significant improvement in the BBB scores was observed in the low-dose group compared to the SCI group (Figure 2(a)). The BBB score in the high-dose group reached a functional plateau at 8 weeks postsurgery at approximately 6.4, significantly higher than the score 3.1 of 1.9 in the low-dose and SCI groups, respectively (*n* = 7 rats per group, *p* < 0.01).

To further determine the functional status of the severed spinal cord, we analyzed the MEPs at 8 weeks postsurgery. In the high- and low-dose groups, after a 25 mA stimulation was applied at the cranial level, we detected an electrical signal at the tibialis anterior muscle. However, no signal was detected in the SCI group (Figure 2(b)). To compare the MEP recovery of the high- and low-dose groups, we quantitatively analyzed the amplitude and latency of the MEPs. We found that the signals in the high-dose group exhibited greater amplitudes and shorter latencies than those in the low-dose group (Figures 2(c) and 2(d)). Taken together, these data further confirm the superior functional recovery achieved in the high-dose group.

### 3.3. Abundant NF-Positive Neuronal Fibers Regenerated at the Injury Site in the High-Dose Group

**Figure 3:**
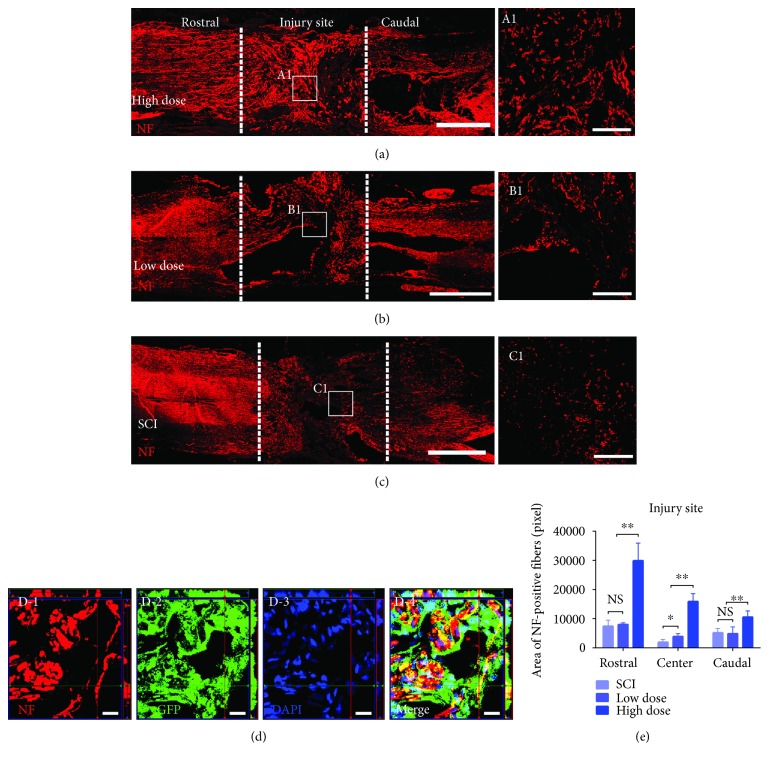
Abundant NF-positive neuronal fibers regenerated at the injury site in the high-dose group. (a–c) Representative images for longitudinal tissue section from the injury site with NF immunostaining showing NF-positive fibers regenerating in the high-dose, low-dose, and SCI group at 8 weeks postsurgery. Scale bars: 1 mm. (A1, B1, and C1) High magnification images from (a–c) showing NF-positive fibers in the center of injury areas (the white dotted lines indicated the boundary between the host spinal cord and the lesion area). Scale bars: 200 *μ*m. (D-1, D-2, D-3, and D-4) Confocal images with z-stack revealing grafted GFP-expressing NSPC-derived NF-positive fibers in the high-dose group. Scale bars: 20 *μ*m. (e) 8 weeks after surgery, quantitative analyses of the number of NF-positive fibers in the rostral, center, and caudal to the lesion area. Note that more NF-positive fibers appear to penetrate and occupy the entire area of the injury site in the high-dose group than the low-dose and SCI group, especially the central area of the injury site (A1), while seldom appear in the corresponding region in the low-dose group (B1) and even almost no in the corresponding region in the SCI group (C1). Besides, the number of NF-positive fibers at the central of the injury site in the low-dose group is higher than the SCI group (mean ± SEM, ^∗^*p* < 0.05, ^∗∗^*p* < 0.001. NS: not significant, *n* = 4 animals per group).

Many previous studies have demonstrated that only a few neuronal fibers regenerate at the injury site following complete SCI, especially in the central region of the injury site [37]. To confirm whether high-dose NSPC transplantation increased the distribution of neuronal fibers at the injury site, we used longitudinal tissue sections containing the injury site from the three groups to stain for NFs. The results showed greater NF-positive fiber penetration and occupation in the entire lesion area in the high-dose group than in the low-dose and SCI groups, notably in the lesion core (Figures 3(a)–3(c)). We further quantitatively analyzed the number of NF-positive fibers in the rostral, central, and caudal regions of the lesion area. The data showed a significantly enhanced distribution of NF-positive fibers in the rostral, central, and caudal regions of the lesion in the high-dose group compared with the other two groups (Figure 3(e)). Interestingly, there were more NF-positive fibers in the central region of the lesion in the low-dose group than in the SCI group, although NF-positive fibers were rarely observed in the central region (Figure 3(e)). Furthermore, by triple-immunostaining for NF, GFP, and DAPI, we demonstrated that most of the NF-positive fibers in the lesion area were derived from grafted GFP-expressing NSPCs (Figure 3(d)).

### 3.4. High-Dose NSPC Transplantation Significantly Increased the Expression of Neural Marker MAP-2 at the Spinal Cord Transection Site

**Figure 4:**
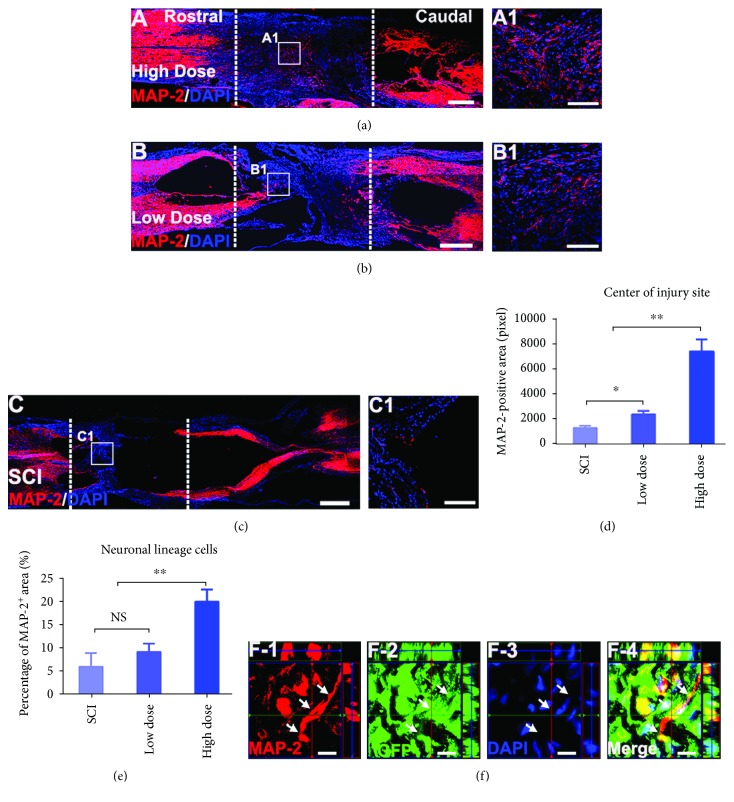
High transplantation dose significantly increased the distribution of MAP-2-positive neurons in the spinal cord transection site. (a–c) Overview of MAP-2 and DAPI fluorescent immunolabeling in a horizontal section showing excellent MAP-2-positive neurons filling in the T10 complete transection site in the high-dose group compared to the low-dose and SCI group at 8 weeks postgrafting (the white dotted lines indicated the boundary between the host spinal cord and the lesion area). Scale bars: 500 *μ*m. (A1, B1, and C1) Higher magnification from (a–c) revealing MAP-2-positive neurons distribute in the central of the injury site. Scale bars: 200 *μ*m. (d) Quantitative analyses of the area of MAP-2 positive in the central of the lesion area for all groups at 8 weeks postsurgery. Note that the transplantation of NSPCs can increase the area of MAP-2 positive compared to the SCI group. Meanwhile, the area of center in the injury site was significantly greater in the high-dose versus low-dose group (mean ± SEM, *n* = 4 animals per group; ^∗^*p* < 0.05, ^∗∗^*p* < 0.01). (e) Quantitative analyses of the percentage of the MAP-2-positive area in the total area of lesion site that is the sum of MAP-2, GFAP, and MBP-positive area at injury site in each subject for three groups (mean ± SEM, *n* = 4 animals per group; ^∗^*p* < 0.05, ^∗∗^*p* < 0.01). (F-1, F-2, F-3, and F-4) A z-stack image triple labeled for GFP, MAP-2, and DAPI reveals that GFP-expressing NSPC-derived MAP-2-positive neurons in the injury site (white arrow indicating the MAP-2-positive neuron extend two dendrite). Scale bars: 10 *μ*m.

To test whether enhancing the NSPC transplantation dose increased the distribution of neurons at the injury site, we applied MAP-2 and DAPI fluorescence immunolabeling to a horizontal spinal cord section from each of the three groups. Representative images showed a significant difference in the distribution of MAP-2-positive neurons between the high- and low-dose groups and between the NSPC transplantation groups and the SCI group over the entire lesion area, notably in the center (Figures 4(a)–4(c)). Quantitative analyses showed an increased MAP-2-positive area in the central region of the lesion in the NSPC transplantation groups compared with the SCI group. Importantly, the MAP-2-positive area in the center of the injury site was significantly greater in the high-dose group than in the low-dose and SCI groups (Figure 4(d)). In addition, we chose four subjects from each group for a quantitative analysis of the proportion of the MAP-2-positive area of the total lesion area, which was the sum of the injury site areas positive for MAP-2, GFAP, and MBP, in each subject at 8 weeks postsurgery. The data revealed a significantly improved percentage of neurons in the lesion tissue in the high-dose group compared with the low-dose and SCI groups (Figure 4(e)). However, we also found that the percentage of neurons in the lesion tissue was not enhanced in the low-dose group compared with the SCI group, although the distribution of MAP-2-positive neurons in the central region of the lesion was increased (Figure 4(d)). By immunolabeling with GFP, MAP-2, and DAPI, we demonstrated the presence of many MAP-2-positive neurons derived from GFP-expressing grafted NSPCs (Figure 4(f)).

### 3.5. Grafted NSPC-Derived Oligodendrocytes Contributed to the Remyelination of Growing Axons in the High-Dose Group

**Figure 5:**
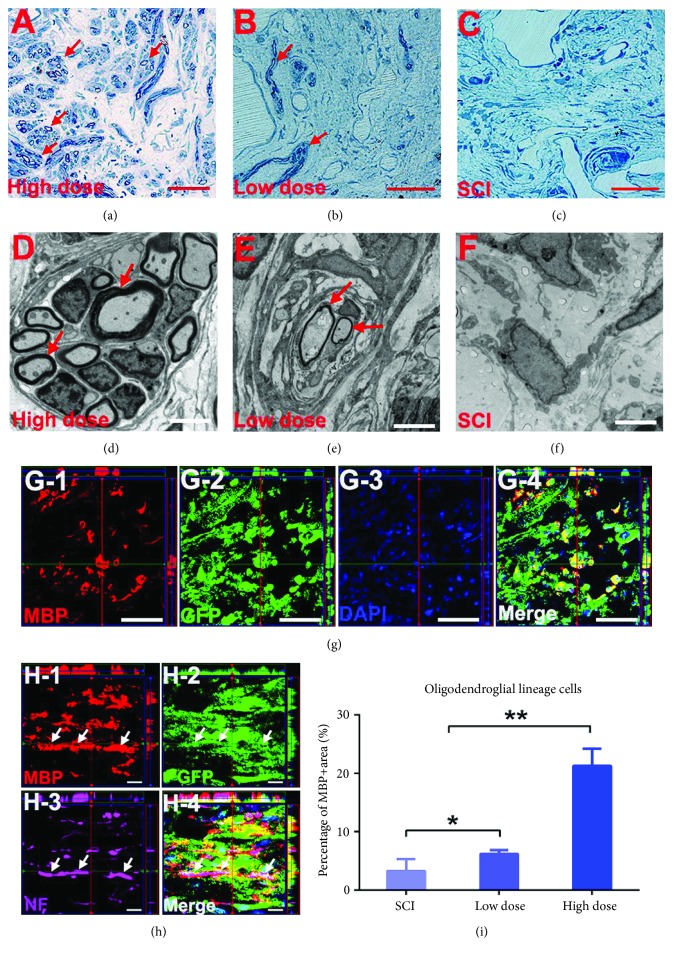
Grafted NSPC-derived oligodendrocytes contributed to remyelination of regenerated axons in the high-dose group. (a–c) Representative images from the injury site showing a great quantity and a small quantity of remyelination, respectively, in the high-dose and low-dose group (arrowheads) but no remyelination in the SCI group. Scale bars: 50 *μ*m. (d, e) TEM reveals myelination of axons occurred in the injury/graft site in the high-dose and low-dose group (arrowheads). Scale bars: 4 *μ*m. (f) TEM revealed the formation of glial scar at the lesion area in the SCI group. Scale bars: 4 *μ*m. (G-1, G-2, G-3, and G-4) Representative images of axial sections stained for MBP, GFP, and DAPI revealed some grafted GFP-positive NSPCs differentiate into MBP-positive oligodendrocytes. Scale bars: 50 *μ*m. (H-1, H-2, H-3, and H-4) A representative z-stack image quadruple labeled for GFP, MBP, NF, and DAPI, indicating GFP+/MBP+ double-labeled myelin sheaths were observed around GFP+/NF+ neuronal fibers (arrowheads). Scale bars: 10 *μ*m. (i) Quantitative analyses of the percentage of the MBP-positive area in the total area of the lesion site that is the sum of MAP-2, GFAP, and MBP-positive area at the injury site in each subject for three groups (values are mean ± SEM, *n* = 4 animals per group; ^∗^*p* < 0.05, ^∗∗^*p* < 0.01).

In our study, more myelination was observed by light microscopy at the spinal cord transection site in the high-dose group than in the low-dose group (Figures 5(a)–5(c)). Using TEM analysis, we detected many myelinated fibers regenerated in the high-dose group and few myelinated fibers regenerated in the low-dose group; almost no regenerated myelinated axons were observed in the lesion area in the SCI group (Figures 5(d)–5(f)). These results suggest that some engrafted cells differentiated into mature oligodendrocytes, forming thick sheaths to wrap growing axons at the SCI site in the NSPC transplantation groups, as recent reports have indicated that neural progenitor cells grafted into lesions can contribute to remyelination [39]. Therefore, we utilized GFP, MBP, and DAPI immunolabeling at the SCI graft site, and the immunohistochemical analyses revealed many GFP/MBP double-positive oligodendrocytes in the lesion area (Figure 5(g)). Based on these results, we added NF staining and observed some GFP/MBP double-positive myelination of the GFP/NF double-positive fibers (Figure 5(h)). Furthermore, for each group, we quantified the proportion of MBP-positive cells in the total area positive for three cell type-specific markers in the lesion. Of the regenerated tissue, approximately 21.3% of the area was positive for MBP in the high-dose group, and this value was significantly higher than that for the low-dose (6.2%) and SCI groups (3.2%). Surprisingly, statistical analysis showed a significant difference between the low-dose (6.2%) and SCI groups (3.2%), meaning that low-dose NSPC transplantation also appeared to induce oligodendrocyte differentiation and remyelination (Figure 5(i)).

### 3.6. High-Dose NSPC Transplantation Significantly Alleviated Glial Scar Formation at the Injury Site

**Figure 6:**
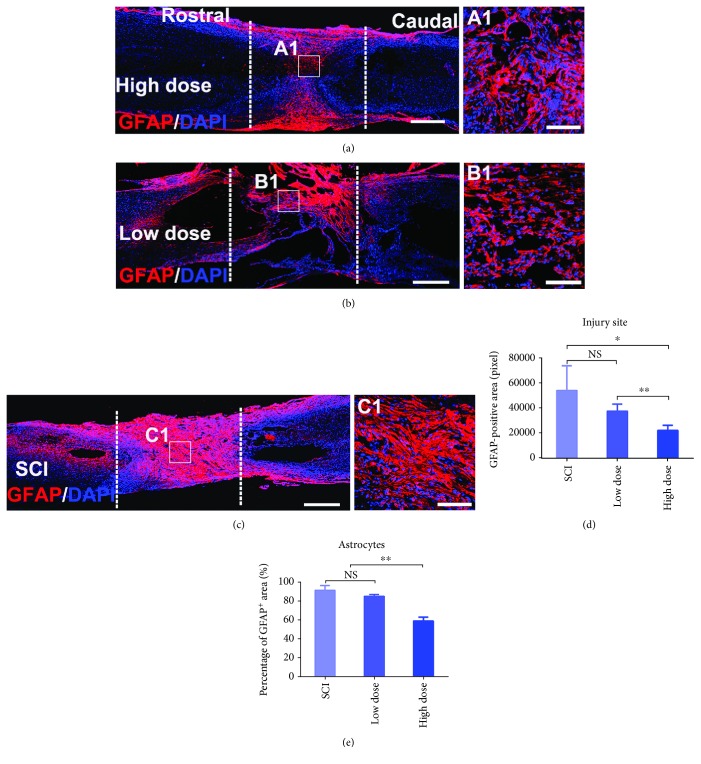
High-dose NSPC transplantation significantly alleviated glial scar formation in the injury site at 8 weeks postgrafting. (a–c) Representative horizontal sections stained for GFAP and DAPI from three groups, (a) revealing moderate GFAP-positive astrocytes distribute in the lesion area in the high-dose group. But there are very dense and strong GFAP-positive astrocytes that fill in the lesion area in the low-dose and SCI group (b, c) (the white dotted lines indicated the boundary between the host spinal cord and the lesion area). Scale bar: 500 *μ*m. (A1, B1, and C1) High-magnification views of (a–c) showing GFAP-positive astrocytes in the central of the lesion area. Scale bar: 200 *μ*m. (d) Quantitative analyses of the GFAP-positive area in the lesion area (mean ± SEM, *n* = 4 animals per group; ^∗^*p* < 0.05, ^∗∗^*p* < 0.01). (e) Quantitative analyses of the percentage of the GFAP-positive area in the total area of the lesion site that is the sum of MAP-2, GFAP, and MBP-positive area at the injury site in each subject for three groups (mean ± SEM, *n* = 4 animals per group; ^∗^*p* < 0.05, ^∗∗^*p* < 0 .01).

We also observed glial scarring and the formation of a cavity in the lesion area. However, a remarkably different phenomenon was observed in the high-dose group at 8 weeks postgrafting. At 8 weeks postgrafting, immunohistochemical analysis of GFAP revealed that the GFAP-positive astrocyte area was decreased over the whole lesion area in the high-dose group compared with the low-dose group and especially with the SCI group (Figures 6(a)–6(c)). To investigate whether the glial scar reduction was associated with the high-dose NSPC transplantation, four subjects from each group were sacrificed for quantification of the GFAP-positive astrocyte area in the lesion. As expected, the results showed significantly less GFAP-positive glial scarring in the lesion in the high-dose group. In contrast, massive glial scarring was observed in the low-dose group, with no significant difference from that observed in the SCI group (Figure 6(d)). In addition, we quantified and analyzed the proportion of GFAP-positive astrocytes in the regenerated tissue structure at the injury site. The proportion (only 58.8%) was significantly lower in the high-dose group than in the low-dose (84.7%) and SCI groups (90.9%) (Figure 6(e)).

### 3.7. Grafted NSPC-Derived Neurons Formed a Nascent Functional Neural Network in a Relayed Manner

**Figure 7:**
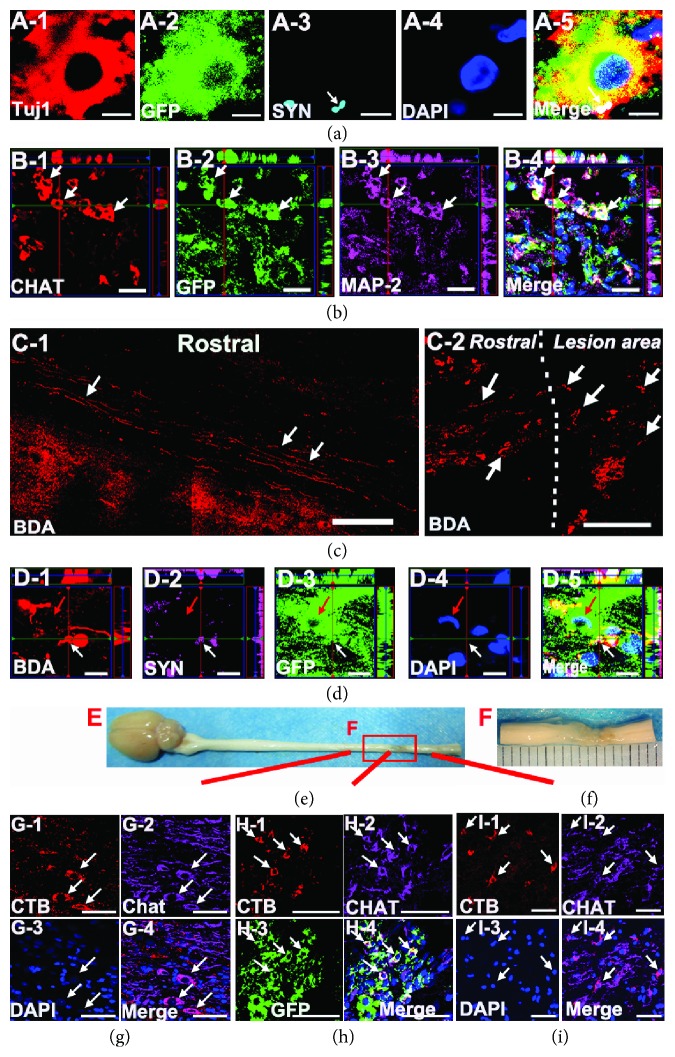
BDA-labeled fibers in the lesion area and grafted NSPC-derived neurons formed a nascent functional neural network in a relayed manner at 8 weeks postgrafting in the high-dose group. (A-1, A-2, A-3, A-4, and A-5) Immunographs showed a graft-derived, GFP-labeled, and Tuj1-positive neuron that express a synaptic marker (synaptophysin (SYN), white arrow) at the lesion area. Scale bars: 4 *μ*m. (b) quadruple immunolabeling for CHAT (choline acetyltransferase), MAP-2, GFP, and DAPI, indicating some grafted NSPC-derived neurons also expressed CHAT, characteristic of spinal motor neurons (arrow). Scale bars: 20 *μ*m. (C1) Sagittal view of BDA-labeled CST axons in the rostral host spinal cord of the lesion site in the high-dose group (white arrow). Scale bars: 200 *μ*m. (C2) Sagittal view of the rostral host-injury border (dashed lines) from a rat in the high-dose group, depicting a few BDA-labeled CST fibers regenerated in the rostral border of the lesion area (white arrow). Scale bars: 100 *μ*m. (D-1, D-2, D-3, D-4, and D-5) A z-stack image showed the formation of synaptic-like structures (SYN, white arrow) between BDA-labeled CST fibers and GFP-expressing grafted cell (red arrow) in the rostral host-injury border. Scale bars: 10 *μ*m. (E) Representative image of gross anatomical changes in the lesion areas of the high-dose group at 8 weeks after the operation. (F) High magnitude image from (E) indicating apparent regenerated tissues in the lesion area. 8 weeks postsurgery, b-CTB was injected into the L2/L3 spinal cord to label CST axons. 1 week after injection, immunostaining revealing that CTB was detected in CHAT-positive motor neurons at the T7-T8 spinal cord segments (G-1, G-2, G-3, and G-4); meanwhile, we detected CTB in the CHAT-positive grafted NSPC-derived motor neurons at the injury site (H-1, H-2, H-3, and H-4) as well as the CHAT-positive motor neurons in the caudal host spinal cord of the injury site (I-1, I-2, I-3, and I-4) (arrow). Scale bars: 50 *μ*m.

Indeed, we observed more functional recovery and neuronal tissue regeneration in the high-dose group than in the other two groups. To further explore the underlying mechanism relating the functional recovery and the abundant neuronal tissue regeneration, we investigated whether functional structure formation occurred at the injury site. At 8 weeks after high-dose NSPC grafting, the immunolabeling analyses revealed that many grafted NSPC-derived neurons expressed a characteristic marker of spinal motor neurons, CHAT (Figure 7(b)). In addition, we also observed a graft-derived, GFP-labeled, and Tuj1-positive neuron expressing a synaptic marker of synaptic junctions at the lesion area, suggesting synaptic connectivity among the grafted neurons (Figure 7(a)). To confirm whether CST fibers were regenerated across the injury site in the high-dose group, 10% BDA was injected into eight sites per hemisphere to trace corticospinal axons at 8 weeks postsurgery. Two weeks later, the immunohistochemical analyses revealed that no BDA-positive CST fibers had regenerated across the injury site, and only some BDA-labeled fibers at the rostral border of host-graft interface were detected. Interestingly, we observed the formation of synaptic-like structures between the BDA-labeled CST fibers and GFP-expressing grafted cells at the rostral host-injury border based on immunostaining for GFP/BDA/SYN/DAPI (Figure 7(d)). Altogether, these results suggested that grafted GFP-labeled cells formed synaptic connectivity with grafted cells and with the host spinal cord.

As nascent synaptic networks were developed at the injury site in the high-dose group, we next investigated whether the nascent synaptic networks could deliver signals across the transection site. Biotinylated CTB, a bacterial toxin for neuronal tracing, can transfer across synapses to second- and third-order neurons, allowing functional neural networks to be traced in the spinal cord. At 8 weeks postgrafting, 1% CTB was injected into the spinal cord of subjects at the L2/L3 level. One week later, the subjects were sacrificed. By multiple immunostaining, we found that CTB was clearly present in caudal CHAT-positive spinal motor neurons and passed through neuronal fibers and arrived at spinal motor neurons at the T8/T9 level. Notably, CTB was also detected in grafted GFP-expressing NSPC-derived CHAT-positive motor neurons at the lesion core in the high-dose group (Figures 7(g)–7(i)). In the SCI group, CTB was almost entirely absent in the lesion area and in the rostral spinal cord over the injury site.

## 4. Discussion

Previous studies have indicated that only a few transplanted NSPCs can survive and most of the survived cells differentiate into astrocytes due to the absence of extracellular matrix support and the formation of a detrimental microenvironment in severe SCI [23, 27]. Recent reports have emphasized the importance of transplantation dose to the survival, migration, and differentiation of grafted NSPCs and functional recovery in mice with moderate SCI [28]. However, to the best of our knowledge, the effect of transplantation dose on donor cell engraftment, lineage-specific integration, and the harmful microenvironment has not been characterized in an acutely transected SCI. In our study, we transplanted a low dose (2.5 × 10^5^) and a high dose (1.5 × 10^6^) of NSPCs into the site of complete SCI immediately after transection. By analyzing the differentially expressed genes among the high-dose, low-dose, and SCI groups in the microenvironment during the acute phase of SCI, we found that the grafted NSPCs in the high-dose group decrease the extent of the inflammatory response (TGF-*β*1, IL-10) and enhance the levels of secreted factors (BDNF, IGF-1, GDNF, and VEGF-A) compared with the SCI group. But there are no difference between the SCI group and the low-dose group. Among the differentially expressed genes, TGF-*β*1 and IL-10 play critical roles in alleviating inflammatory injury and preventing neural apoptosis [15, 40]. BDNF, IGF-1, GDNF, and VEGF-A contribute to neuronal survival, axonal plasticity, and synaptic function, including neurotransmitter availability [41, 42]. This result indicated that high-dose NSPCs can improve harmful microenvironment in the lesion area by neuroprotective and immunomodulatory effects in the acute phase of severe SCI. Meanwhile, we demonstrated that the extent of donor cell engraftment is positively correlated to the transplantation dose in severe SCI rats at 1 week posttransplantation. We can supposedly say that grafted cell survival was associated with the microenvironment in the lesion area, such as the extent of the inflammatory reaction and the levels of secreted neurotrophic factors.

At 8 weeks postsurgery, similar to the findings of a previous report, we found that grafted NSPC differentiation was mainly restricted to the glial lineage [6, 43, 44]. Meanwhile, we observed that the high transplantation dose markedly enhanced the neuronal cell and oligodendrocyte distribution in the entire lesion area. However, in the low-dose group, the neuronal cell distribution was increased only at the lesion core compared with the SCI group. Besides, we quantified the proportion of MAP-2-positive neurons at the lesion area and found that the proportion of MAP-2-positive neurons in the high-dose group is significant higher than the low-dose and SCI group. These data suggested that there is a positive linear relationship between the total distribution of mature neurons and the transplantation dose at the lesion area. The explanation for this phenomenon may be related to the higher survival of grafted NSPCs which enhance the number of neurons and the improvement of the harmful microenvironment which contribute to endogenous NSPC migration into the lesion area and differentiation into neurons [8, 15].

In addition, many studies have reported that extensive astrocytic scar formation in the lesion area inhibits the fibers regenerated and the function recovery in rodents living for long periods after complete SCI [45, 46]. In our study, we demonstrated the high-dose NSPC transplantation significantly decreased scar formation. In the past, many studies have reported alleviation of the formation of glial scar can promote the NF-positive axons regenerated [4, 14, 46, 47]. So we analyze the axons regenerated in the lesion area postgrafted at 8 weeks and found that significantly increased NF-positive axons distributed throughout the entire lesion area in the high-dose group compared with the low-dose group and the SCI group. Using GFP and NF immunostaining, we detected grafted NSPC-derived NF-positive axon and axons extended from the host spinal cord in the lesion area. We believe that this finding is important due to regenerated axons that may provide a structural basis for the reestablishment of neural signal transduction across the lesion area. However, we did not detect BDA-labeled CST regenerate through the lesion by BDA anterograde tracer in our study. Other studies have also reported the same result and thought that CST stumps are remote from the neuronal cell body and vulnerable to pathological insults, as well as the lack of environment supporting the CST fibers regenerated in the lesion area, which may cause difficulties for CST regeneration [48, 49].

The significantly increased distribution of oligodendrocytes and myelinated fibers in the lesion area also was detected in the high-dose group. By using NSPC-GFP, we identified a large number of oligodendrocytes derived from grafted NSPCs and GFP-expressing NSPC-derived MBP-positive oligodendrocytes which can form thick sheaths wrapping growing axons in the lesion area. This notion is consistent with previous publication suggesting the transplantation of NSPCs contributes to remyelination [50]. Notably, the formation of myelinated fibers has obvious functional benefits, promoting electrophysiological signal transduction passing through the injury site [39, 51, 52].

In previous observations, NSPC transplantation had limited effects on the functional recovery in severe SCI [19]. But we found most of these studies applied relatively low doses and ignored the large cavity in the lesion area, which caused a poorly engraftment of the grafted cells. However, in the present study, we observed dose-related changes in open-field locomotor performance in severe SCI (250,000 versus 1,500,000 NSPCs). At 2 weeks postgrafting, the high-dose groups exhibited significantly higher BBB scores than the SCI group. Notably, 5 weeks later, the high-dose group was superior to the low-dose group in open-field locomotor performance. This may be explained by the massive distribution of neurons and the axon regenerated in the lesion area in the high-dose group. A parallel investigation of the relationship between the transplantation dose and functional recovery revealed MEP recovery at 8 weeks postsurgery in the high-dose and low-dose groups but not in the SCI group, and the recovery of latency and amplitude in the high-dose group is significant better than the low-dose group. Because there were significantly higher NF-positive axons distributed throughout the entire lesion area in the high-dose group compared with the low-dose group, spontaneous axonal regeneration in the SCI group is limited [46, 53].

In the CNS, the disruption of functional connections is the primary cause of sustained dysfunction following SCI. In 2010, Abematsu et al. has demonstrated that the abundant neuronal differentiation of NSPCs is crucial of functional recovery after SCI [26]. Then, Lu et al. has reported that the mechanism of recovery is functional to relay formation after severe SCI [54]. More recently, several lines of evidence have suggested that newly born neurons form a nascent functional neural network participate in locomotor functional recovery after severe SCI [37, 55, 56]. In the present study, the immunohistochemical and behavioral analyses revealed that the high-dose NSPC transplantation promoted neuronal distribution and integration, resulting in abundant axonal regeneration and functional recovery. We extensively studied the roles of the neurons and growing axons responsible for functional recovery using two neuronal tracers, TEM, and multiple immunofluorescence staining. The results showed that while no CST fibers regenerated across the lesion area except some BDA-labeled CST fibers that regenerated at the border of host-graft interface, synapses were formed with grafted cells, and a certain amount of renascent medullated fibers integrated into the lesion site, which dramatically accelerated signal transduction [39]. Besides, our study also found that CTB which was injected into the L2/L3 white matter reached transplant-derived motor neurons at the lesion core and crossed the injury site to enter the rostral host motor neurons, further confirming the formation of functional synapse-containing neuronal pathways in the high-dose group [26].

## 5. Conclusions

High-dose transplantation can remarkably contribute to NSPC survival and neuronal distribution and can increase the proportions of neurons and oligodendrocytes in the lesion area. The increased grafted NSPC-derived motor neurons and extensive regenerated axons can form a nascent functional neural network, which significantly promoted functional recovery in the high-dose group. It is important to consider potential dose-related effects on donor cell survival, neuronal distribution, and locomotor recovery in the development of preclinical NSPC transplantation therapy for severe SCI.

## Figures and Tables

**Table 1:** Primers used in this study.

Gene symbol	5′-Forward primer-3′	5′-Reverse primer-3′
BDNF	TTAGCGAGTGGGTCACAGCGG	CGAGTTCCAGTGCCTTTTGTCTATG
IL-6	GATTGTATGAACAGCGATGATGC	AGAAACGGAACTCCAGAAGACC
VEGF-A	CCAGGCTGCACCCACGACAG	TCATTGCAGCAGCCCGCAC
NGF	TGCATAGCGTAATGTCCATGTTG	TGTGTCAAGGGAATGCTGAAGT
GDNF	AGAGGGAAAGGTCGCAGAGG	GCTTCACAGGAACCGCTACAAT
NT3	GACACAGAACTACTACGGCAACAG	ACTCTCCTCGGTGACTCTTATGC
IL-1b	CCCAACTGGTACATCAGCACCTCTC	CTATGTCCCGACCATTGCTG
TNF-*α*	GGGCTCTGAGGAGTAGACGATAAAG	GGGCAGGTCTACTTTGGAGTCATTG
IL-10	GACAACATACTGCTGACAGATTCCT	TCACCTGCTCCACTGCCTTG
TGF-*β*1	TGCGCCTGCAGAGATTCAAG	AGGTAACGCCAGGAATTGTTGCTA
IGF-1	GGCACCACAGACGGGCATTG	AGGCTTCAGCGGAGCACAGTACAT
GAPDH	TGGAGTCTACTGGCGTCTT	TGTCATATTTCTCGTGGTTCA

## Data Availability

The datasets used and/or analyzed during the current study are available from the corresponding author on reasonable request.

## Abbreviations

NSPCs: Neural stem/precursor cells
SCI: Spinal cord injury
BBB score: Basso-Beattie-Bresnahan locomotor rating scale
GFP: Green fluorescent protein
MAP-2: Microtubule-associated protein-2
Tuj1: *β*III tubulin
SYN: Synaptophysin
NF: Neurofilament
GFAP: Glial fibrillary acidic protein
MBP: Myelin basic protein
CHAT: Choline acetyltransferase
DAPI: 4′,6-diamidino-2-phenylindole
MEPs: Motor-evoked potentials
CST: Corticospinal tract fibers
BDA: Biotinylated dextran amine
CTB: Biotinylated cholera toxin B subunit.

## Supplementary Materials

Supplementary Materials Characterization of neural stem/progenitor cells (NSPCs) from the GFP transgenic rat in vitro and in the lesion at 8-week posttransplantation.
