# Supplementary material for: High-Dose Neural Stem/Progenitor Cell Transplantation Increases Engraftment and Neuronal Distribution and Promotes Functional Recovery in Rats after Acutely Severe Spinal Cord Injury
Source: Stem Cells Int. 2019 Sep 2;2019:9807978. doi: 10.1155/2019/9807978 (PMC6745168; doi:10.1155/2019/9807978)
Supplement: Supplementary Materials — Characterization of neural stem/progenitor cells (NSPCs) from the GFP transgenic rat in vitro and in the lesion at 8-week posttransplantation. [file 9807978.f1.docx]

Supplementary Materials

Characterization of neural stem/progenitor cells (NSPCs) from the GFP transgenic rat in vitro and GFP-expressing NSPC-derived NeuN positive neurons, O1 positive oligodendrocytes and GFAP positive astrocytes in the lesion at 8-week post transplantation.


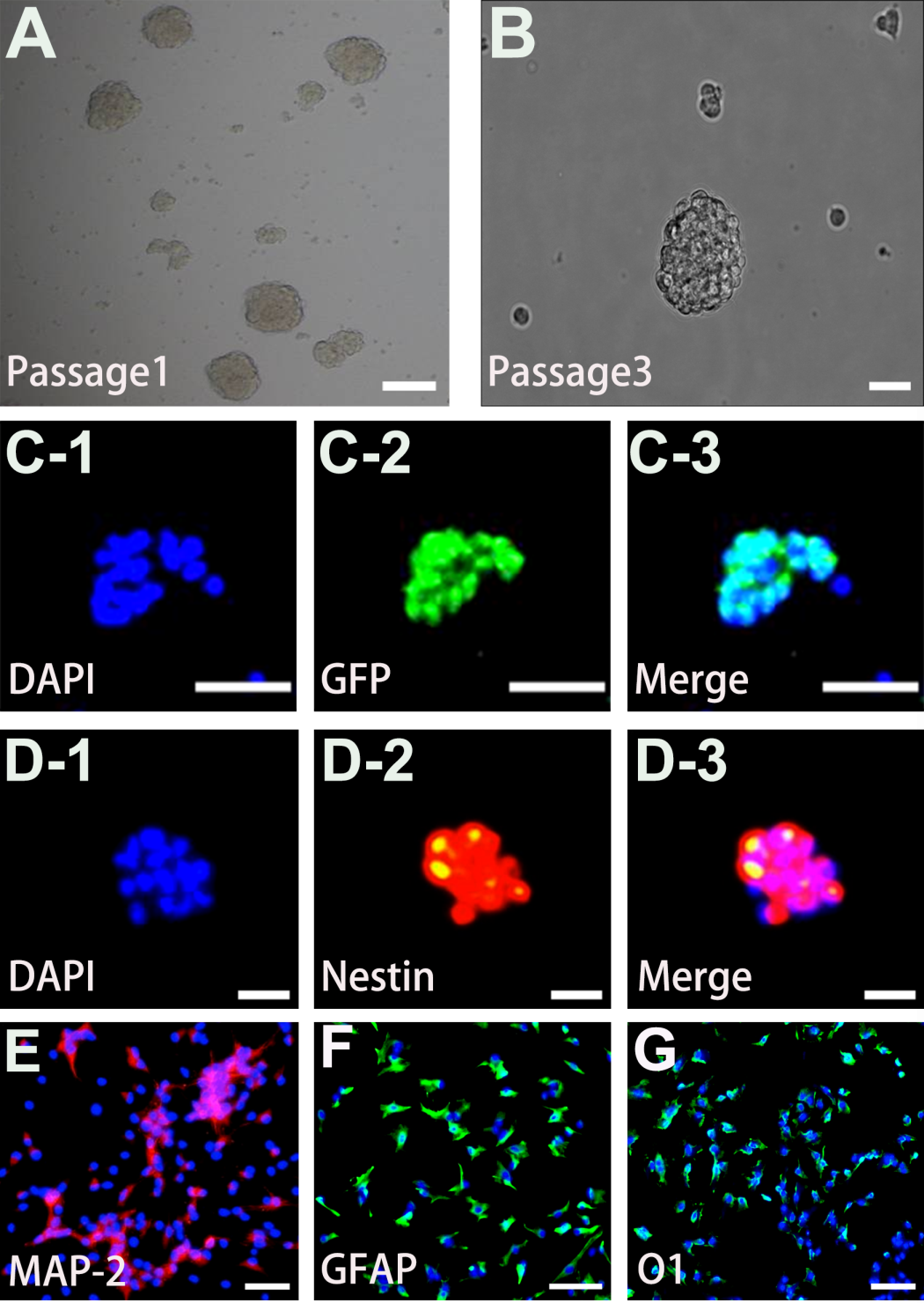


Figure Supplement 1. Characterization of neural stem/progenitor cells (NSPCs) from the

GFP transgenic rat in vitro and the differential potential of NSPCs in culture.(A) (B)

respectively showing the NSPCs in passage 1 and passage 3. Scale bars: 100 um. (C)

immunofluorescent staining with DAPI showing neurosphere-expressed GFP in passage

3. Scale bars: 100 um. (D) Immunofluorescent staining with DAPI and Nestin showed

neurosphere-expressed nestin in passage 3 before transplantation. Scale bars: 100 um.

(E) immunofluorescent staining of the neuronal marker MAP-2 revealed the neural

differentiation of NSPCs in vitro. (F) immunofluorescent staining of the astroglial marker

GFAP. (G) immunofluorescent staining of the oligodendrocyte marker O1. Scale bars: 100

um.


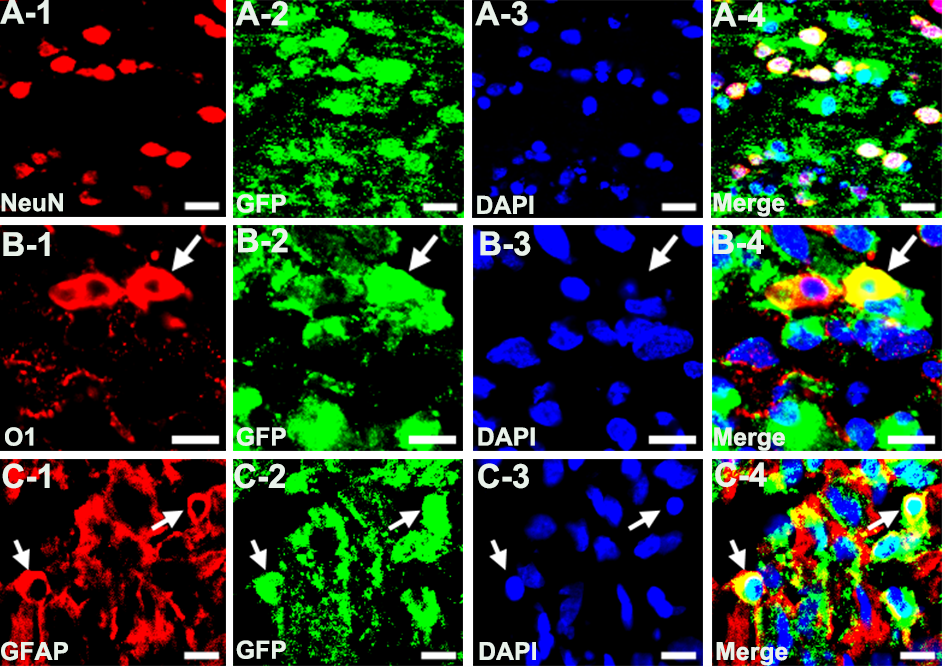


Figure Supplement 2. Immunostaining respectively showed grafted GFP-expressing NSPC-derived NeuN positive neurons (A), O1 positive oligodendrocytes (B) and GFAP positive astrocytes (C) in lesion area at 8-week post-surgery. Scale bars: 10 um.
